# PEDOT:PSS in Water and Toluene for Organic Devices—Technical Approach

**DOI:** 10.3390/polym12030565

**Published:** 2020-03-04

**Authors:** Beata Jewłoszewicz, Krzysztof A. Bogdanowicz, Wojciech Przybył, Agnieszka Iwan, Ireneusz Plebankiewicz

**Affiliations:** Military Institute of Engineer Technology, Obornicka 136 Str., 50-961 Wroclaw, Poland; jewloszewicz@witi.wroc.pl (B.J.); przybyl@witi.wroc.pl (W.P.); iwan@witi.wroc.pl (A.I.); plebankiewicz@witi.wroc.pl (I.P.)

**Keywords:** PEDOT:PSS, organic devices, spin coating, doctor blade coating, spray coating, thermographic camera

## Abstract

Poly(3,4-ethylenedioxythiophene:poly(styrenesulfonate) (PEDOT:PSS) water and toluene solutions were investigated in detail, taking into consideration their stability, wettability, transparency, and electrochemical properties, along with change polarity caused by dopant. As dopant, methanol, ethanol, and isopropanol were used with different dipole moments (1.70, 1.69, and 1.66 D) and dielectric constants (33.0, 24.5, and 18.0). Three techniques, i.e., spin coating, doctor blade coating, and spray coating, were employed to created PEDOT:PSS layers on glass, glass/indium tin oxide (ITO), and glass/fluorine-doped tin oxide (FTO) substrates with optimized technical parameters for each used equipment. All used PEDOT:PSS water and toluene solutions demonstrated good wetting properties with angles below 30° for all used surfaces. Values of the energy bandgap (Eg) of PEDOT:PSS investigated by cyclic voltammetry (CV) in solution showed increase energy Eg along with addition of alcohol to the mixture, and they were found in the range of 1.20 eV to 2.85 eV. The opposite tendency was found for the Eg value of the PEDOT:PSS layer created from water solution. The storage effect on PEDOT:PSS layers detected by CV affected only the lowest unoccupied molecular orbital (LUMO) level, thereby causing changes in the energy bandgap. Finally, simple devices were constructed and investigated by infrared (IR) thermographic camera to investigate the surface defects on the created PEDOT:PSS layers. Our study showed that a more stable PEDOT:PSS layer without pin-holes and defects can be obtained from water and toluene solutions with isopropanol via the spin coating technique with an optimal speed of 3000 rpm and time of 90 s.

## 1. Introduction

In recent years, increased interest in organic devices, such as solar cells (SCs), was observed in both academia and industry, focusing on high efficiency and solution-based fabrication [1,2,3]. Comparing different configurations of polymer solar cells (PSCs), a classification into mesoscopic (n-i-p) PSCs, planar (n-i-p) PSCs, and inverted planar (p-i-n) PSCs can be made [4,5,6]. Considering the typical planar architecture, the addition of a mesoscopic layer complicates the manufacture process, while also requiring a high-temperature sintering operation, which is not compatible with polymer-based flexible substrates [7]. Hence, the currently trending roll-to-roll industrially available mass production of planar or inverted planar SCs could benefit from flexible and low-temperature solution processes [8].

Flexible and low-temperature processes are needed to create transparent electrodes, which are important for many optoelectronic devices, i.e., touch screens, organic light-emitting diodes, and solar cells [7]. A transparent and flexible electrode should possess some characteristics such as optical transparency, high conductivity, and, in some cases, a high level of bending toughness without a significant decrease in electrical performance. A golden balance needs to be found between the electrical resistivity and transmittance, since good conductivity does not align well with transparency. At the moment, commercial transparent electrodes are based mainly on indium tin oxide (ITO). However, its poor mechanical properties, its relatively low conductivity in flexible transparent electrodes, and the scarcity of indium necessitate the search for a new generation of optoelectronic devices [9].

Generally, strategies for the preparation of flexible transparent electrodes can be classified as follows:

(1) conducting polymers (e.g., poly(3,4-ethylenedioxythiophene:poly(styrenesulfonate) (PEDOT:PSS), polyaniline (PANI), polypyrrole (PPy)) [10,11,12];
(2) inorganic nanostructures (e.g., carbon nanotubes, graphene, metal nanowires) [13,14,15,16];
(3) hybrid materials (e.g., single-walled carbon nanotubes, l,d-PLA, PANI nanowire/nylon nanofiber) [17,18].

In organic devices, other layers in addition to the electrodes should also possess good flexibility and transparency. It is well known that PEDOT:PSS can act as a transparent electrode in devices, while it can also play the role of a hole transporting layer (HTL) in organic solar cells, taking into consideration its high work function, high transparency, and good conductivity. Unfortunately, PEDOT:PSS in organic solar cells exhibits various disadvantages when it comes to HTL formation, with a consequent decrease in the long-term stability and performance of devices [19]. The main disadvantages are as follows: (i) aggregation of particles in a water solution of PEDOT:PSS, (ii) degradation of ITO surface, (iii) instability of the ITO/PEDOT:PSS interface in devices due to strong acid characteristics of PSS in PEDOT:PSS, (iv) hydrophilic characteristics of PEDOT:PSS solution, and (v) instability of PEDOT:PSS in water in a short time.

To solve these difficulties, various inorganic compounds such as V_2_O_5_, WO_3_, NiO, and MoO_3_ were proposed instead of PEDOT:PSS applied in organic solar cells [20]. Organic compounds used as HTLs in organic solar cells mainly include poly(styrene sulfonic acid) grafted with polyaniline (PSSA-*g*-PANI) [19], polypyrrole–polystyrene sulfonate (PPy:PSS) [21], PEDOT:PSS and amphiphilic surfactant Surfynol 104 series [20], PEDOT:PSS with mixed Ag and Au nanoparticles (dual NPs) [22], or a new polymer with carbazole and PO_3_ moieties (PC-P) [23].

In recent years, poly(3,4-ethylenedioxythiophene:poly(styrenesulfonate) (PEDOT:PSS), especially with regard to its use in electronic devices including photovoltaics, received interest on account of its relatively simple processability at low temperature, antireflecting properties, and water-based dispersion [24,25,26,27]. In some devices, water-free PEDOT:PSS is required, mainly due to the design of the layer deposition process via evaporation from toluene solution or the vulnerability of some components to moisture, i.e., in the construction of perovskite solar cells [28,29,30].

PEDOT:PSS can be also used as a hybrid electrode over ITO or fluorine-doped tin oxide (FTO) giving improved electrical properties and mechanical support [31,32,33], or acting as a hole transporting layer (HTL) in dye sensitizing and perovskite solar cells [34,35].

PEDOT:PSS was investigated in the past taking into consideration both molecular and supramolecular engineering concepts. In this study, we would like to present a technical approach in the preparation of PEDOT:PSS layers for organic devices via an investigation of layer formation via spin coating, doctor blade coating, and spray coating techniques. Our main focus was on the comparison of different PEDOT:PSS solutions based on pure solvents like water or toluene and their mixtures with different alcohols such as methanol, ethanol, and isopropanol. In this article, we evaluate the electrical, thermal, and electrochemical properties of thin PEDOT:PSS layers over three different substrates (glass, glass/ITO, and glass/FTO) in order to assess the influence of alcohol adjuncts on conductive polymers. As a substrate, we tested glass with ITO or FTO, taking into consideration the fact that, depending on the type of organic solar cells, ITO or FTO substrate is mainly used. In organic/polymer solar cells, ITO substrate is mainly used. In DSSCs, FTO substrate is mainly used, while, in perovskite solar cells, both ITO and FTO were tested to find the best conductive substrate.

In our work, for the first time, a thermographic camera was used to detect the location of defects in the created devices, as well as to determine the electrical behavior of PEDOT:PSS and its mixture with alcohols.

In this work, we tested commercially available PEDOT:PSS in water or toluene solution. Our aim was to investigate how three alcohols with different polarity can influence the selected properties of PEDOT:PSS, taking into consideration a ternary component solution. As alcohols, methanol, ethanol, and isopropanol were used with different dipole moments (1.70, 1.69, and 1.66 D) and dielectric constants (33.0, 24.5, and 18.0). Our study showed that the presence of isopropanol with a lower dipole moment and lower dielectric constant in PEDOT:PSS solution used for layer preparation caused an almost two-fold increase in the electrical resistance and a 30 °C decrease in the thermal response.

## 2. Materials and Methods

### 2.1. Materials

All chemical reagents and substrates were purchased from a commercial source. The mainly used components were a PEDOT:PSS (0.5:0.8 weight ratio) aqueous dispersion with a solid content of 1.3 wt.% (Sigma-Aldrich, Saint Louis, MO, USA), PEDOT:PSS dispersion in toluene with a solid content of 1.5 to 2.5 wt.% (M125 HTL Solar 3, Ossila, Sheffield, UK), and high-purity alcohols, such as methanol, ethanol, and isopropanol. To study PEDOT:PSS layers, we used the following substrates: glass, FTO- and ITO-coated glass. Microscope slides (20 × 20 × 1 mm, ChemLand, Stargard Szczecinski, Poland ) were used for UV–Vis spectrometry. For FTO (20 × 15 × 2.2 mm, unpatterned, Ossila, Sheffield, UK), we used cyclic voltammetry for the electrochemical study with an IR thermographic camera ITO (20 × 15 × 1.1 mm, edgeless 8 pixel, Ossila, Sheffield, UK).

### 2.2. Preparation of PEDOT:PSS Solutions

**Figure 1:**
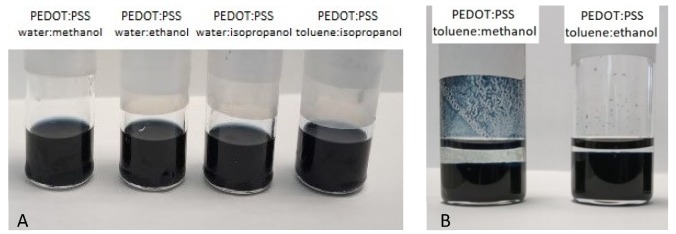
Poly(3,4-ethylenedioxythiophene:poly(styrenesulfonate) (PEDOT:PSS) in water or toluene mixed with different alcohols: methanol, ethanol, and isopropanol ((**A**)—stable mixtures, (**B**)—unstable mixtures).

PEDOT:PSS solutions dispersed in water or toluene were mixed with three different alcohols, methanol, ethanol, or isopropanol with volume ratio of 1:0.5. All mixtures were ultrasonicated for 1 h at room temperature. PEDOT:PSS mixtures in toluene with methanol and ethanol were unstable after 30 min, at which point they stratified (Figure 1A). For these reasons, we investigated in detail only four mixtures: PEDOT:PSS in water with all three alcohols and PEDOT:PSS in toluene with isopropanol (Figure 1B). All mixtures were prepared for tests directly before film application, while, for aging tests, they were stored in the fridge.

### 2.3. Preparation of PEDOT:PSS Films

PEDOT:PSS films were prepared by spin coating, doctor blade coating, and spray coating techniques. All substrates (glass, glass/FTO, glass/ITO) were cleaned alternately with isopropanol and acetone, three times, prior to use. Additionally, all substrates were placed in UV Ozone Cleaner for 10 min. The layers for each mixtures were prepared with the same set of parameters as for pristine PEDOT:PSS solutions. Films deposited by spin coater were prepared at 3000 rpm for 90 s. Layers made using the doctor blade device were applied at a speed of 50 mm/s, and the casting knife was set at 10 µm. In the case of samples prepared by spray coater, the solution was sprayed on the substrate from a height of 10 cm with air pressure of 2 bar. All prepared films were annealed on a hot plate at 30 °C for 20 min.

### 2.4. Methods

A contact angle goniometer (Ossila, UK) coupled with a computer system served as the basic diagnostic tool that enabled contact angle measurements via drop shape analysis after wetting the surface with aqueous or toluene solutions. The drop images were recorded up to 5 s counting from the moment the drop touched the surface. The volume of a drop dosed with a syringe was on the order of 7 μL. As the contact angle, we used the angle between the tangent to the surface of the drop placed on the solid at the point of contact of three phases: solid, liquid, and gas.

The transmission UV–Vis spectra were acquired using an A360 UV–Vis spectrophotometer (AOR Instruments, Shanghai, China) with an interval of 0.2 nm and medium scan speed.

Electrochemical measurements were carried out as described in our previous work [36], using a Metrohm Autolab PGSTAT M204 potentiostat (Barendrecht, Nederland) and an electrochemical cell containing a glassy carbon electrode (diameter 2 mm), a platinum rod, and Ag/AgCl as working, counter, and reference electrodes, respectively. Potentials are referenced with respect to ferrocene (Fc), which was used as the internal standard. Cyclic voltammetry experiments were conducted in a standard one-compartment cell, in acetonitrile (Honeywell, Charlotte, N.C., USA, ≥99.9%), under argon atmosphere. Furthermore, 0.2 M Bu_4_NPF_6_ (Alfa Aesar, Haverhill, MA, USA, 99%) was used as the supporting electrolyte. The concentration of compounds was equal to 1.0 × 10^−6^ mol/dm^3^. The deaeration of the solution was achieved by argon bubbling through the solution for about 15 min prior to the measurement. All electrochemical experiments were carried out at ambient temperature and pressure.

Thermal behavior was observed according to our standard protocol described elsewhere [17], using a thermographic camera (VIGOcam v50, VIGO System S.A, Ożarów Mazowiecki, Poland) while applying a bias voltage between 0 and 10 V and using a multichannel potentiostat–galvanostat (PGStat Autolab M101, Metrohm, Barendrecht, Nederland) connected to a computer. The experiment was designed to apply voltage in a programmed way. The potential was applied in a range from 0 V to 10 V with 0.5-V steps between different values over 3 min for each voltage. The current response was recorded during 3-min intervals, and each step was separated with a 10-s window; when the IR image was collected, the value of the applied potential of the current step was maintained. Both the camera and the power source were controlled via computer software.

## 3. Results and Discussion

In this study, we investigated in detail a technical approach in the preparation of PEDOT:PSS layers for organic devices and their influence on optical and electrochemical properties. Our main focus was on the comparison of different PEDOT:PSS solutions based on pure solvents like water or toluene and their mixtures with three different alcohols. We performed an assessment of the wettability of different surfaces with selected solutions and their impact on the preparation of thin layers via spin coating, spray coating, or doctor blade techniques. Moreover, an evaluation of the electrochemical behavior of PEDOT:PSS in solution or in a layer was also performed.

### 3.1. Contact Angle Measurements

One of the most crucial factors to be taken into account when spreading a solution is the wettability of solid substrates. The contact angle is one of the commonly used methods to assess the wettability of a surface or material. Obtained results give a direct answer of whether a liquid applied over a solid surface has the tendency to exhibit good wetting properties. Usually, in this case, the contact angle is smaller than 90°. In the opposite situation, for non-wetting liquids, the contact angle is between 90 and 180° [37]. In this study, we evaluated water- and toluene-based PEDOT:PSS solutions and the influence of the addition of alcohols such as methanol, ethanol, and isopropanol on the wetting properties of glass, glass/ITO, and glass/FTO surfaces. We focused only on the wettability aspect, which could be investigated in a simple way, giving a general comparison viewpoint for different layer preparation techniques.

#### 3.1.1. PEDOT:PSS in Water without and with Alcohols

The results of static contact angle measurements for aqueous PEDOT:PSS solutions with and without the addition of alcohols are summarized in Table 1. All used solutions demonstrated good wetting properties with angles below 30° for all used surfaces, as expected. The static contact angles for glass and ITO-coated glass surfaces ranged around 24–26° and 19–23°, respectively, within the experimental error for all solutions, except for the mixture with isopropanol. The difference in the behavior, namely, the increase in hydrophobic nature, giving values reaching 30°, can be a result of the lower polarity of water:isopropanol with respect to pure aqueous solution and other alcohols used in this study. In the case of FTO-coated glass, the contact angle increased in the order water:methanol ≤ water < water:ethanol < water:isopropanol, giving values of 19.7°, 22.6°, 24.0°, and 25.3°, respectively. For this substrate, the solution with the addition of methanol gave the lowest average contact angle from the series. However, taking into account the value deviation, the value was very similar to the pure aqueous solution.

#### 3.1.2. PEDOT:PSS in Toluene without and with Alcohols

A similar experiment was also performed for toluene-based PEDOT:PSS solutions with and without the addition of alcohol. In these cases, the solutions demonstrated good wetting properties with angles below 30° for all used surfaces, as expected. It was noticed that solutions containing methanol and ethanol formed emulsions and suffered from low stability, resulting in separation over time. The separation was relatively fast and occurred in the syringe used for contact angle measurements; therefore, contact angle measurements for PEDOT:PSS in toluene:methanol and toluene:ethanol were not recorded. The results of wettability are presented in Table 2. The toluene-based solution with and without isopropanol displayed very similar behavior, resulting in the surfaces having decreasing wettability in the order ITO-coated glass < glass < FTO-coated glass.

### 3.2. PEDOT:PSS Layer Preparation Via Three Different Techniques

In this section, PEDOT:PSS layers were prepared via three techniques, namely, spin coating, doctor blade, and spray coating. The application parameters for individual devices were selected during the initial optimization process. The focus of this section was on the evaluation of three different film-forming techniques in order to select the best one that gives reproducible results using PEDOT:PSS water- or toluene-based solutions with or without the addition of alcohol.

#### 3.2.1. Spin Coating

**Figure 2:**
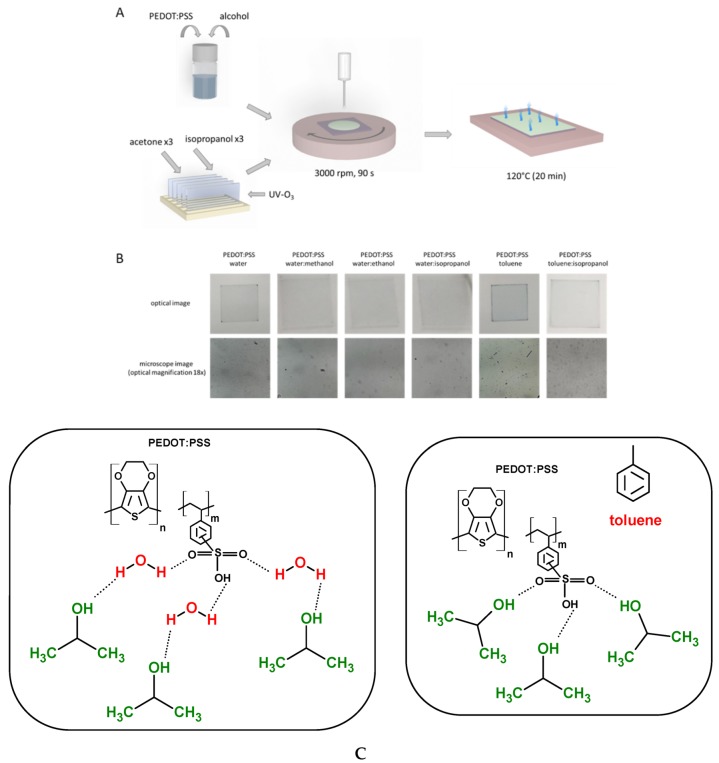
Scheme of layer preparation using spin coating technique (**A**); images of PEDOT:PSS layers (spin coated at 3000 rpm, 90 s) and microscopic images acquired at optical magnification of 18× (**B**); scheme of primary and secondary hydrogen bond interactions of PEDOT:PSS in water and toluene with isopropanol (**C**).

As the first step of this work, selected parameters were changed to optimize the spin coating technique. We changed the speed value from 2000 to 4000 rpm and time from 30 to 90 s until we found the best technical parameters for the PEDOT:PSS solutions (Figure 2A). Details of the optimization of the spin coating technique for PEDOT:PSS in water and toluene are presented in Supplementary Materials (Figures S1 and S2).

In our case, for the spin coater, the optimal speed value was found to be 3000 rpm for 90 s. The layer obtained in such conditions was estimated to have 80 nm thickness, based on the spin speed to thickness correlation provided by Ossila. Due to the specific limitations of this technique, it was possible to obtain repeatable samples for both water- and toluene-based solutions. Layers of PEDOT:PSS with and without alcohols obtained via spin coating were more homogeneous (see Figure 2B). In Figure 2B (lower row), the microscopic images show in more detail the reduction in the presence of small spots for samples prepared from the pure solution and with the addition of alcohol. There was no difference between pure solutions or between different alcohols present in the mixture. Based on our understanding, the improvement in layer formation due to the presence of isopropyl alcohol can be related to the improved interaction between polymer and solvent. The advantage of isopropanol over other tested alcohols is related to its chemical structure and the longer hydrocarbon chain, as schematically presented in Figure 2C. In the case of the water solution of PEDOT:PSS, primary hydrogen bond interactions existed between the hydrogen atom of water and the oxygen atoms in SO_3_H groups of the PSS polymer. Moreover, H–H interactions cannot be excluded. In the case of the toluene solution of PEDOT:PSS, these reactions did not take place. In both solutions of PEDOT:PSS, interactions between the alcohol and OH or SO_3_H groups are possible, as schematically presented in Figure 2C. It should be stressed that, in the case of PEDOT:PSS in the water solution, alcohol was used as a secondary dopant because water was the first one. In the case of the toluene solution of PEDOT:PSS, alcohol was the primary dopant. Moreover, it should be stressed that the most spectacular changes in morphology of created layers were found for layers with isopropanol as a dopant, probably because of the symmetry of this alcohol and steric hinderance.

#### 3.2.2. Doctor Blade Technique

Selected parameters were also changed to optimized the process of layer formation for the doctor blade technique. We changed the gap value as 10, 25, and 50 µm and speed rate as 20, 50, and 80 mm/s to find the best technical parameters for PEDOT:PSS solutions. Details of the optimization of the doctor blade technique for PEDOT:PSS in water and toluene are presented in Supplementary Materials (Figures S3 and S4).

**Figure 3:**
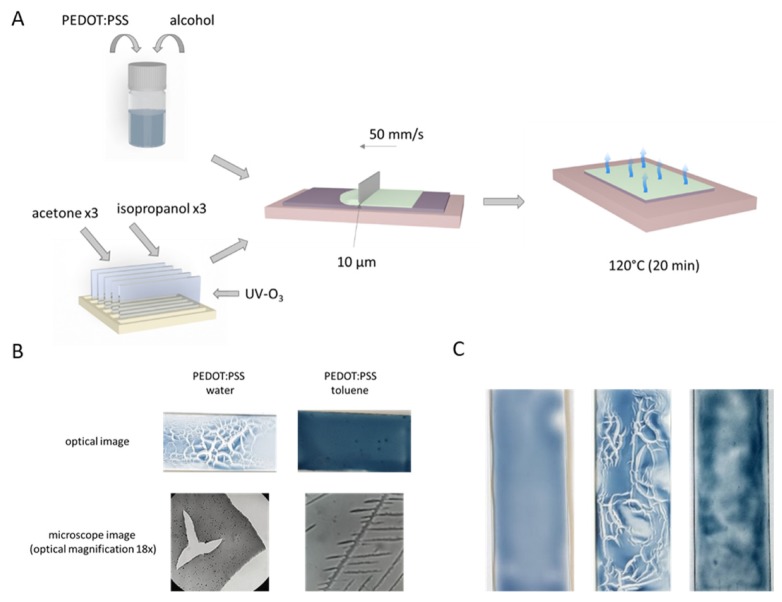
Scheme of layer preparation using doctor blade technique (**A**); images of PEDOT:PSS layers (10 µm gap, 50 mm/s) and microscopic images acquired at optical magnification of 18× (**B**); PEDOT:PSS (from water) layer (from left to right): directly after casting, after annealing, and layer made from PEDOT:PSS (from water:isopropanol) layer after annealing (**C**).

PEDOT:PSS layers were applied with a doctor blade device with a gap set at the 10-µm layer with 50 mm/s film developing speed (Figure 3A). Formation of layers using this technique was quite problematic. The problematic part was the aqueous or toluene solution’s tension while spreading the solution on the surface. Moreover, in the case of the aqueous layer applied on the surface after annealing, crack formation resulted over the whole layer (Figure 3B,C, middle image). The addition of alcohol was beneficial for a reduction in surface tension, and the formed layer did not crack; however, the homogeneity aspect was not met.

In the case of toluene-based solutions after annealing, a visually more homogenous layer was formed (see Figure 3B, top right). The PEDOT:PSS layer under 18× magnification revealed the formation of long crystalline patterns present in layers formed in the mixtures with alcohols (Figure 3B, bottom right). It seems that a thick layer was formed due to the liquid surface tension, which would be unacceptable for fine organic devices.

#### 3.2.3. Spray Coating

In the case of spray coating, we changed the distance between spray and substrate from 6 to 14 cm to create the best PEDOT:PSS layers. Details of the optimization of the spray coating technique for PEDOT:PSS in water and toluene are presented in Supplementary Materials (Figures S5 and S6).

**Figure 4:**
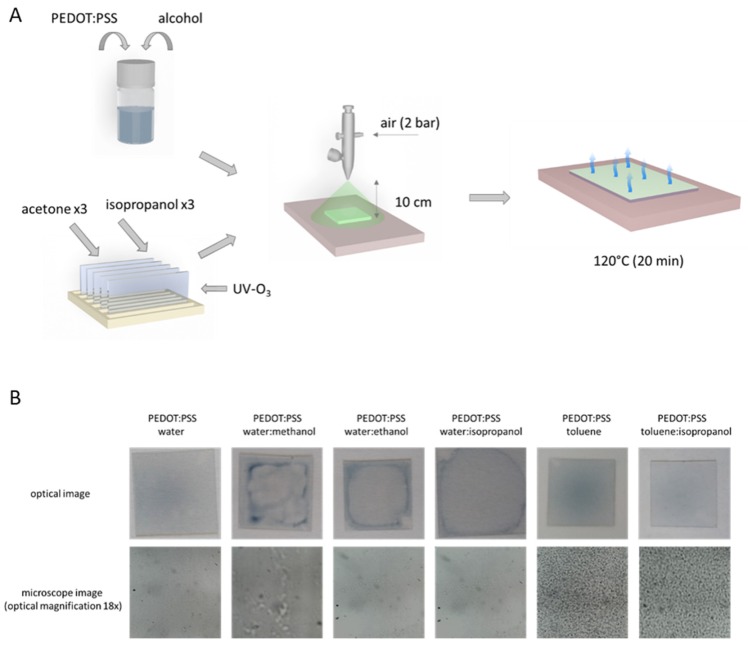
Scheme of layer preparation using spray coating technique (**A**); images of PEDOT:PSS layers (spray coated, 10 cm distance, pressure 2 bar) and microscopic images acquired at optical magnification of 18× (**B**).

The last technique used was a spray coating set-up composed of an airbrush connected to a Metabo Basic 250-24W compressor. The layers were sprayed from a height of 10 cm at an air pressure of 2 bar (Figure 4A). This technique had a similar problem to the doctor blade technique regarding the tension of used solvents. The principle of this technique is the formation of droplets, and their accumulation forms the final layer. Spraying aqueous-based solutions resulted in the formation of uneven layers due to the cone-shaped stream, where the center was more likely to receive more solution. This technical issue can easily be avoided by using a more precise device for solution application. However, the PEDOT:PSS layers from water, water:methanol, and water:ethanol where inhomogeneous, whereas layers made from water:isopropanol (Figure 4B, top row) showed the formation of relatively homogenous layers, as confirmed by optical microscopy (Figure 4B, bottom row).

The layers prepared from toluene-based PEDOT:PSS both with and without isopropanol addition gave better layers in terms of visual quality compared to aqueous solutions. The biggest disadvantage was the formation of small agglomerates observed under the optical microscope (magnification 18×), which would hinder the application of these samples. The agglomerate formation is related to the mechanism of layer formation using this technique; during dispersion of the polymeric solution, before the solution gets into contact with the solid surface, it loses solvent via partial evaporation. Due to this fact, in some parts of the formed layer, there were small droplets containing undissolved polymeric material [38]. This effect was observed in the case of toluene-based solutions due to its better volatilization (Figure 4B).

### 3.3. Electrochemical Study

In order to study electrochemical properties of different PEDOT:PSS solutions, cyclic voltammetry (CV) was employed. The first step was to record voltammetry curves for solutions and solvent to identify characteristic signals for single components. The second step involved the electrochemical study of layers on FTO-coated glass, while the third step elucidated the aging process and its influences on the electrochemical response.

#### 3.3.1. CV of PEDOT:PSS in Water and Toluene without and with Alcohols

**Figure 5:**
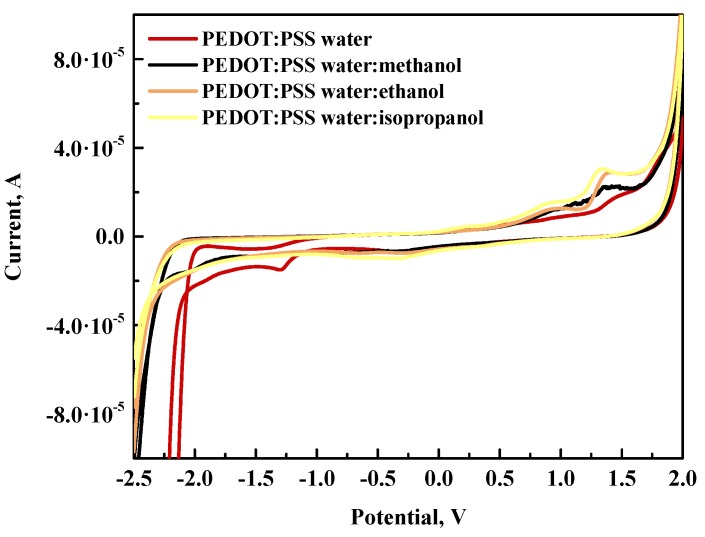

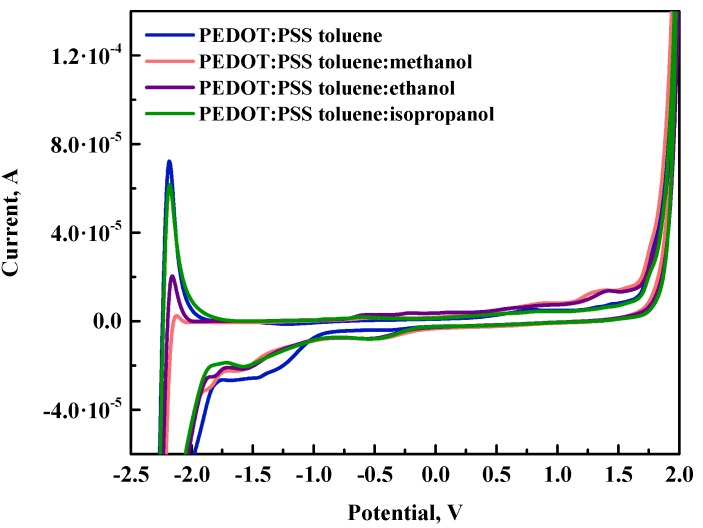
Comparative cyclic voltammetry (CV) curves for PEDOT:PSS in water (top) and toluene (bottom).

The first step involved aqueous and toluene-based PEDOT:PSS solutions, as well as their mixtures with methanol, ethanol, and isopropanol and pure solvents. The cyclic voltammetry response was very stable in the measured range from −2.37 to 0.70 V (see Table 3). In all cases, the response was almost fully reversable, which means that degradation processes were not observed (see Figure 5). The electrochemical response to oxidation conditions for the PEDOT molecule involved acceptance of an electron, which caused rearrangement of the conjugation of double bonds and the formation of a cation and unpaired electron. The second step of oxidation resulted in complete oxidation where only cations are present [39]. It seems that, in the case of water-based solutions, the oxidation to the fully oxidized step occurred in one step, with oxidation onset at 0.38 V. Similar oxidation behavior was observed for the toluene-based PEDOT:PSS solution with oxidation onset at 1.22 V. On the other hand, the reduction of both starting solutions also differed, giving values of offset points at −1.14 V and −1.83 V for PEDOT:PSS in water and toluene, respectively. The difference in redox energies for both solutions was significant in terms of the energy gap (E_g_), and, in this case, the gap was smaller for the toluene-based solution than for the water-based solution by about 0.19 eV. The addition of alcohol to PEDOT:PSS in water resulted in an extension of over 1 eV compared to the starting solution. The best result from the electrochemical point of view was achieved for the water:isopropanol mixture giving an E_g_ value equal to 2.85 eV. The electrochemical series in terms of solvent-containing water based on decreasing E_g_ value was water:isopropanol > water:methanol > water:ethanol > water.

In the case of PEDOT:PSS in toluene, the addition of alcohol also expanded the electrochemical window, giving the highest value for the toluene:isopropanol mixture at 2.54 eV. The results for the mixture with methanol and ethanol gave similar nominal E_g_; however, the values shifted by approximately 0.30 eV toward more negative potentials.

For all samples, additional oxidation maxima at approximately 1.5 V were observed, which can be a consequence of reversible oxidation of some –OH alcohol groups at the platinum electrode [40]. This was also noted in reference measurements performed for pure alcohols.

We based our investigation on commercially available homogenous solutions of PEDOT:PSS in water or in toluene. As a dopant, we used methanol, ethanol, or isopropanol with various dipole moments, as presented in Table 3. It is known that compounds with similar solubility parameters are able to interact with each other, resulting in solvation, miscibility, or swelling. We could conclude that, taking into consideration the dipole moments of the applied alcohols, better solubility should be seen for the water solution of PEDOT:PSS based on the similar values of dipole moments for primary and secondary hydrogen bond interactions.

#### 3.3.2. CV of PEDOT:PSS Layers on Glass/FTO Substrate

In the second step, layers based on PEDOT:PSS solutions in different solvents were prepared via the spin coating method over FTO-coated glass substrate due to the better quality of redox signals registered in the CV curve compared to samples prepared on ITO. In this evaluation, toluene:methanol and toluene:ethanol were not examined due to the instability of solutions. The experimental values of HOMO, LUMO, and energy bandgap are shown in Table 4.

**Figure 6:**
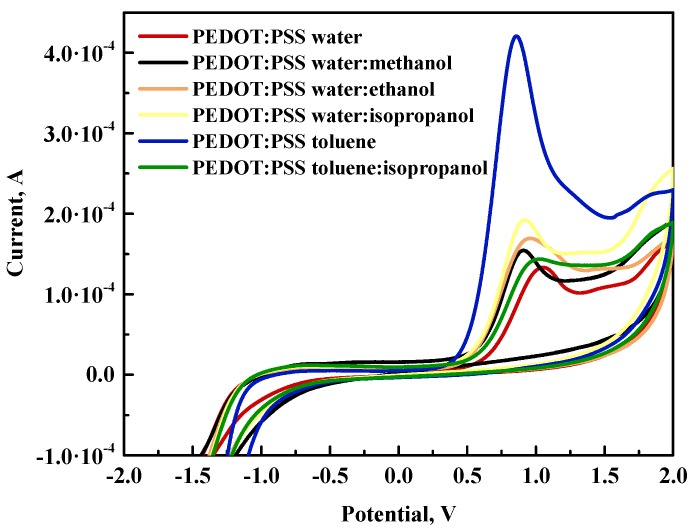
Comparative summary of CV curves for all tested PEDOT:PSS layers.

Generally, upon comparing obtained results for all created layers, it can be easy noted that the HOMO–LUMO limit values were very similar to each other unlike the electrochemical response in solution. This might be an effect of concentration. In solution, the PEDOT:PSS was diluted, whereas, in the form of a thin layer, it was more concentrated. The series of PEDOT:PSS layers depending on used solvent can be organized in terms of decreasing E_g_ value as follows: toluene:isopropanol > water > toluene > water:isopropanol > water:methanol > water:ethanol. The values of energy gap for starting solutions were very similar to those obtained for solutions, where the aqueous solution had a higher value. In the case of layers, the signals for the presence of alcohol in the structure were not observed. In Figure 6, a comparison of all CV curves for PEDOT:PSS layers can be observed. The most visible difference in the spectra was the oxidation signal, which changed depending on the used solution for layer preparation. This might suggest that traces of solvent were present in the formed layer even after annealing at 130 °C. Additionally, the presence of alcohol had a significant impact on the oxidation process occurring in the layer. for the toluene-based solution, isopropanol caused a shift toward positive potentials, increasing the energy bandgap. The opposite situation took place for the water-based solution, where the addition of alcohol caused a shift in oxidation band toward lower potentials, resulting in decreases in E_g_ by 0.22 eV, 0.26 eV, and 0.31 eV for isopropanol, methanol, and ethanol, respectively.

#### 3.3.3. PEDOT:PSS in Water without and with Alcohols in Layers: Storage Effect

In order to evaluate the stability of prepared PEDOT:PSS layers, we carried out short-term storage up to three days in a dark box and argon atmosphere. After each day, the electrochemical performance of the new sample was tested in order to detect any changes in the CV curve. The storage in the abovementioned conditions affected only the LUMO level, thereby causing changes in the energy bandgap (Table 5). It was not possible to find any specific trend for the set of samples. Based on our understanding, the results presented a response to various phenomena such as the evaporation of enclosed solvent or water adsorption from humid air. In our opinion, in order to preserve the original properties of the layer, the best way is to always use a freshly prepared sample.

### 3.4. UV–Vis Study

The hole transport layer (HTL) in organic solar cells such as PEDOT:PSS should be as transparent as possible. We investigated the influence of the addition of alcohols on the transmittance of PEDOT:PSS films. The UV–Vis measurements were carried out for layers made with a spin coater (3000 rpm, 90 s) on a thin glass substrate to avoid the additional influence coming from the thickness of the glass or deposed conductive layers of ITO or FTO. Details on the influence of technical parameters of spin coating on the transmittance spectra are presented in Supplementary Materials (Figures S7 and S8).

**Figure 7:**
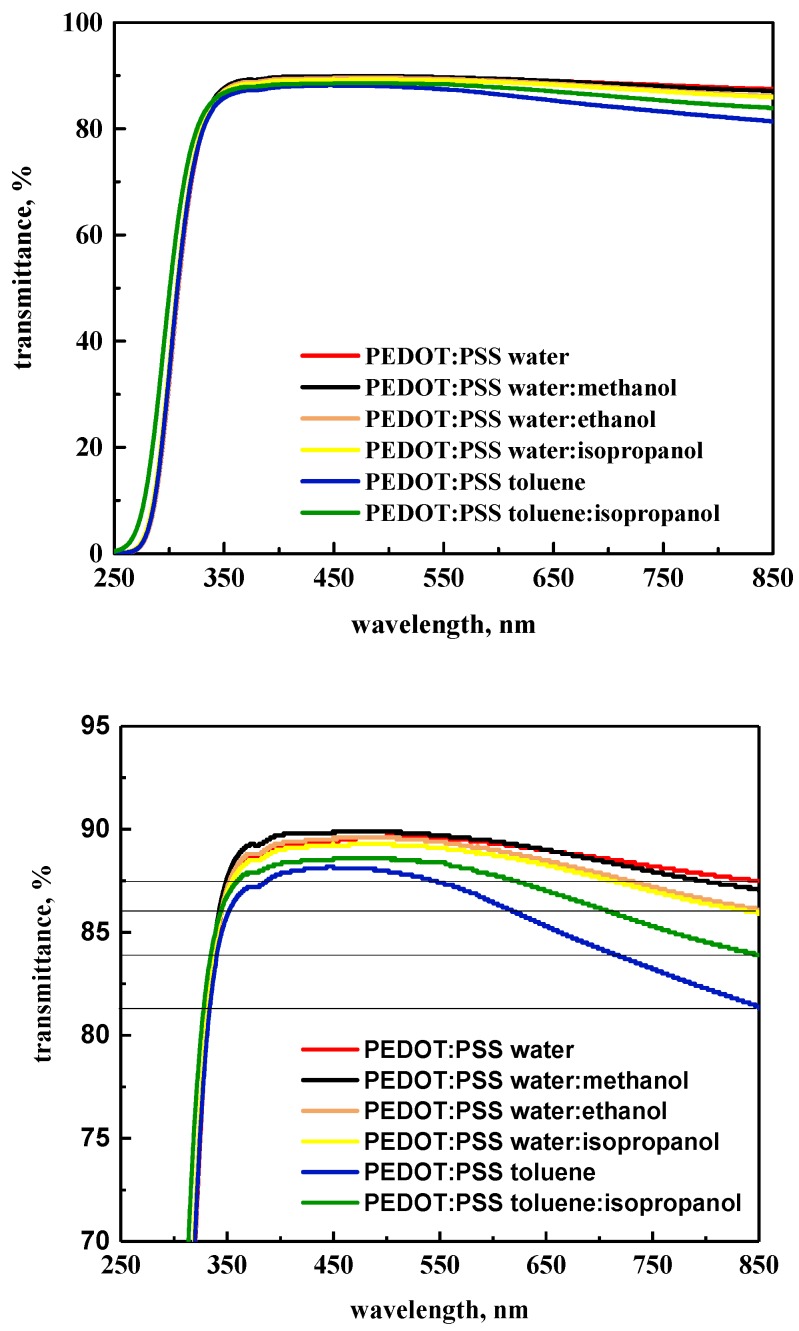
UV–Vis spectra of PEDOT:PSS layers in full (top) and from 70% to 95% (bottom) transition mode.

Figure 7 shows the transmittance spectra for all tested PEDOT:PSS films. At wavelengths larger than 350 nm, the transparency of all films remained at a level of 85%, while they remained at a level of 80% up to 850 nm. On the other hand, for PEDOT:PSS (toluene) films, the value of transmittance dropped slightly to 80%. This means that the addition of alcohols to PEDOT:PSS solutions practically did not affect the transparency of formed layers.

As shown in Figure 7, the type of solvent and dopant influenced changes in the transmittance of the investigated layers. Transmittance for the PEDOT:PSS toluene film mainly dropped in the absorption range from 550 to 850 nm. As explained previously for the water solution, taking into consideration primary and secondary H-bonding, a small drop in transmittance was found due to the dipole moment of alcohol used (a small drop was observed for isopropanol and ethanol used as dopant compared with water and water with methanol). The highest drop was found for the toluene:isopropanol mixture compared with all investigated samples (Figure 7, bottom).

### 3.5. IR Thermography Study

For thermal imaging, four samples (PEDOT:PSS from water, water:isopropanol, toluene, and toluene:isopropanol) were selected. These four samples showed the best electrochemical behavior from the whole series. Thermal imaging was used to observe overall behavior of the sample device during application of an external potential. For this experiment, the active layers were prepared using the spin coating technique over ITO-coated glass support. The architecture of the devices was as follows:

- ITO/PEDOT:PSS(water)/Ag/ITO;
- ITO/PEDOT:PSS(water:isopropanol)/Ag/ITO;
- ITO/PEDOT:PSS(toluene)/Ag/ITO;
- ITO/PEDOT:PSS(toluene:isopropanol)/Ag/ITO.

**Figure 8:**
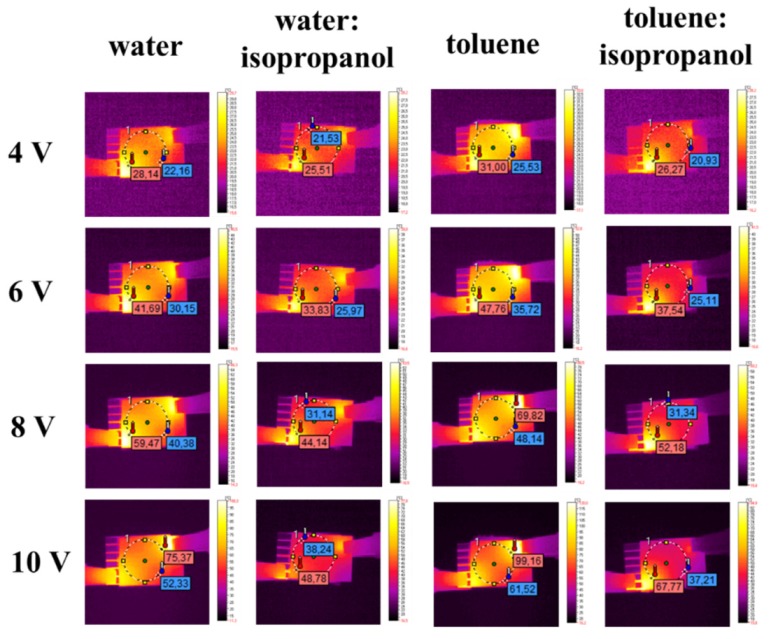
IR images obtained for device architectures (from left to right): ITO/PEDOT:PSS(water)/Ag/ITO, ITO/PEDOT:PSS(water:isopropanol)/Ag/ITO, ITO/PEDOT:PSS(toluene)/Ag/ITO, and ITO/PEDOT:PSS(toluene:isopropanol)/Ag/ITO at 4.0 V, 6.0 V, 8.0 V, and 10.0 V.

**Figure 9:**
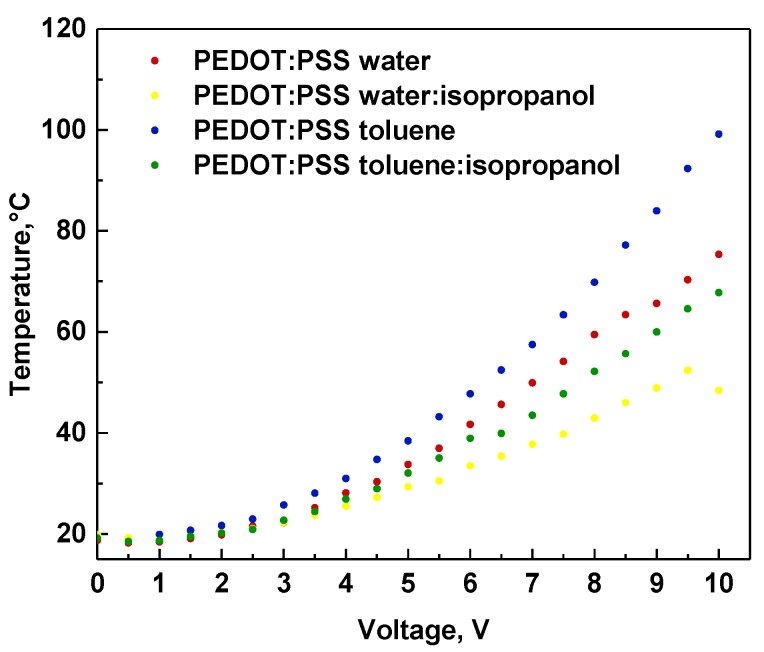
The correlation of temperature versus applied potential for constructed devices containing PEDOT:PSS from water, water:isopropanol, toluene, and toluene:isopropanol.

In Figure 8, IR images are presented for all architectures at selected voltages. In general, for all devices, a very similar pattern of temperature topography was observed. As can be seen in the exemplary images, upon increasing the voltage, two main heating centers located in proximity to both metallic contacts were observed. In the images at 10 V, it is more evident that the heating zone also included the middle part of the active area (see Figure 8, last row). This might suggest that the current flow occurred via the shortest available way. The heat concentration close to the metallic clamps can be explained by the concentration of the current flow close by, as well as due to the imperfect interface between metal and ITO, as observed in our previous work [17,24]. During the experiments, the maximum observed temperature ranged between 40 °C and 100 °C (see Figure 9). The highest temperatures were observed for the ITO/PEDOT:PSS(toluene)/Ag/ITO architecture, whereas the lowest observed temperature was observed for ITO/PEDOT:PSS(water:isopropanol)/Ag/ITO. For samples with the addition of isopropanol, a decrease by about 30 °C in thermal response compared to the corresponding pure solvent was noted. For ITO/PEDOT:PSS(water:isopropanol)/Ag/ITO at 10 V, a small drop in temperature was also noted; however, this was not correlated to any topographic temperature change.

**Figure 10:**
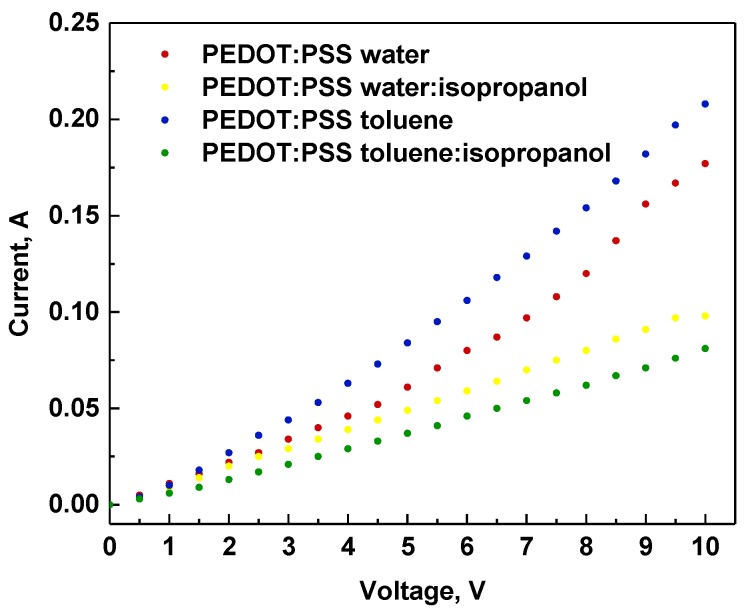
The correlation of current with applied potential for constructed devices containing PEDOT:PSS from water, water:isopropanol, toluene, and toluene:isopropanol.

The analysis of electrical conductivity for all tested samples revealed the behavior of electric conductors correlated with the applied voltage (Figure 10). The resistance values ranged from 45.68 Ω to 121.24 Ω. The results are consistent with the thermal behavior for all samples except for that containing PEDOT:PSS from the toluene:isopropanol solution. In this case, the sample displayed the highest resistance among all samples; however, the temperature observed during current flow was not the highest. This might suggest that part of the electricity was converted to heat. Moreover, a small drop in current at 10 V for ITO/PEDOT:PSS(water:isopropanol)/Ag/ITO was also observed, which influenced the thermal response of the sample.

## 4. Conclusions

To summarize, in our study we presented a technical approach for the preparation of PEDOT:PSS layers for organic devices. Our main focus was on the comparison of different PEDOT:PSS solutions based on pure solvents like water or toluene and their mixtures with three different alcohols. The results of our study demonstrated the following:

- The presence of alcohol did not have a significant influence on the wetting abilities of the prepared solutions for all three tested surfaces (glass, ITO, and FTO);
- In our study, the spin coating technique showed the best reproducibility and homogeneity of prepared samples compared to other techniques on a laboratory scale;
- The preparation of thick layers promotes the crystallization of PEDOT:PSS in the layer;
- The adjustability of E_g_ of layers (mainly in terms of LUMO levels) decreased the starting value in the presence of alcohol;
- The presence of isopropanol in PEDOT:PSS solutions used for layer preparation caused an almost two-fold increase in electrical resistance, as well as a decrease in thermal response by 30 °C.

We can conclude that, for laboratory-scale or small-scale preparation of organic devices, the best technique would be spin coating, which allows the greatest precision in adjusting the parameters. Moreover, the addition of isopropanol allows changing the original behavior of PEDOT:PSS.

## Figures and Tables

**Table 1:** Contact angle results for three different surfaces (glass, glass/indium tin oxide (ITO) and glass/fluorine-doped tin oxide (FTO)) for aqueous-based PEDOT:PSS solutions with and without alcohol.

Sample	Substrate	Average Contact Angle (°)	Image
PEDOT:PSS (water)	Glass	24.2 ± 0.9	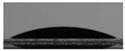
	ITO	19.2 ± 2.5	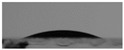
	FTO	22.6 ± 1.2	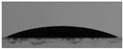
PEDOT:PSS (water:methanol)	Glass	26.9 ± 2.1	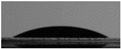
	ITO	23.2 ± 3.3	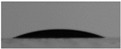
	FTO	19.7 ± 0.9	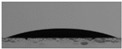
PEDOT:PSS (water:ethanol)	Glass	26.2 ± 2.5	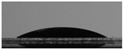
	ITO	22.8 ± 1.2	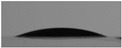
	FTO	24.0 ± 0.8	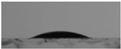
PEDOT:PSS (water:isopropanol)	Glass	30.9 ± 2.5	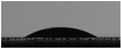
	ITO	28.7 ± 3.5	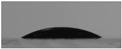
	FTO	25.3 ± 1.6	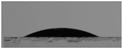

**Table 2:** Contact angle results for three different surfaces (glass, glass/ITO, and glass/FTO) for toluene-based PEDOT:PSS solutions with and without alcohol.

Sample	Substrate	Average Contact Angle (°)	Image
PEDOT:PSS (toluene)	Glass	20.2 ± 0.9	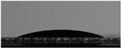
	ITO	18.3 ± 4.2	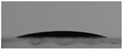
	FTO	20.2 ± 2.0	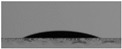
(PEDOT:PSS toluene:isopropanol)	Glass	20.0 ± 3.7	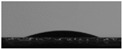
	ITO	16.4 ± 0.4	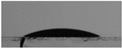
	FTO	20.0 ± 2.1	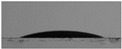

**Table 3:** Summary of selected electrochemical values and dipole moments for aqueous and toluene-based solutions of PEDOT:PSS with and without of alcohol.

Sample (Solutions)	E_HOMO_, eV	E_LUMO_, eV	Energy Bandgap (eV)	Dipole Moments of Dopants (D)
PEDOT:PSS (water)	−4.84	−3.45	1.39	1.85
PEDOT:PSS (water:methanol)	−5.09	−2.75	2.34	1.85:1.70
PEDOT:PSS (water:ethanol)	−5.03	−2.74	2.29	1.85:1.69
PEDOT:PSS (water:isopropanol)	−5.05	−2.20	2.85	1.85:1.66
PEDOT:PSS (toluene)	−4.81	−3.61	1.20	0.39
PEDOT:PSS (toluene:methanol)	−5.32	−3.26	2.06	0.39:1.70
PEDOT:PSS (toluene:ethanol)	−5.64	−3.60	2.04	0.39:1.69
PEDOT:PSS (toluene:isopropanol)	−6.15	−3.61	2.54	0.39:1.66

**Table 4:** Summary of selected electrochemical values for layers prepared from aqueous and toluene based solutions of PEDOT:PSS with and without of alcohol.

Sample (Layers)	E_HOMO_ (eV)	E_LUMO_ (eV)	Energy Bandgap (eV)
PEDOT:PSS from water	−5.05	−3.46	1.59
PEDOT:PSS from water:methanol	−4.98	−3.65	1.33
PEDOT:PSS from water:ethanol	−4.93	−3.65	1.28
PEDOT:PSS from water:isopropanol	−4.97	−3.60	1.37
PEDOT:PSS from toluene	−4.93	−3.41	1.52
PEDOT:PSS from toluene:isopropanol	−5.06	−3.44	1.62

**Table 5:** The results of energy bandgap values for layers after different storage periods.

Sample (Layers)	Day 0	Day 1	Day 2	Day 3
	Energy Bandgap (eV)			
PEDOT:PSS from water	1.59	1.38	1.43	1.77
PEDOT:PSS from water:methanol	1.33	1.54	1.48	1.72
PEDOT:PSS from water:ethanol	1.28	1.42	1.48	1.41
PEDOT:PSS from water:isopropanol	1.37	1.53	1.54	1.57
PEDOT:PSS from toluene	1.52	1.57	1.47	1.46
PEDOT:PSS from toluene:isopropanol	1.62	1.71	1.67	1.45

## Supplementary Materials

The following are available online at https://www.mdpi.com/2073-4360/12/3/565/s1: Figure S1: Photos of PEDOT:PSS (from water) layer created with a speed value from 2000 to 4000 rpm and time from 30 to 90 s from top to bottom, along with images detected by optical microscope with magnification 18×; Figure S2: Photos of PEDOT:PSS (from toluene) layer created with a speed value from 2000 to 4000 rpm and time from 30 to 90 s from top to bottom along with images detected by optical microscope with magnification 18×; Figure S3: Photos of PEDOT:PSS (from water) layer created with the gap set value at 10, 25, and 50 µm and speed rate at 20, 50, and 80 mm/s; Figure S4: Photos of PEDOT:PSS (from toluene) layer created with the gap set value at 10, 25, and 50 µm and speed rate at 50 mm/s; Figure S5: Photos of PEDOT:PSS (from water) layer created by changing the distance between spray and substrate from 6 to 14 cm; Figure S6: Photos of PEDOT:PSS (from toluene) layer created by changing the distance between spray and substrate from 6 to 14 cm; Figure S7: UV–Vis spectra of PEDOT:PSS (from water) layers in transition mode with a speed value from 2000 to 4000 rpm and time from 30 to 90 s from top to bottom; Figure S8: UV–Vis spectra of PEDOT:PSS (from toluene) layers in transition mode with a speed value from 2000 to 4000 rpm and time from 30 to 90 s from top to bottom.
