# Supplementary material for: PEDOT:PSS in Water and Toluene for Organic Devices—Technical Approach
Source: Polymers (Basel). 2020 Mar 4;12(3):565. doi: 10.3390/polym12030565 (PMC7182892; doi:10.3390/polym12030565)
Supplement: Supplementary file 1 [file polymers-12-00565-s001.pdf]

Supporting Information

PEDOT:PSS in water and toluene for organic devices - technical approach

Beata Jewłoszewicz, Krzysztof A. Bogdanowicz*, Wojciech Przybył, Agnieszka Iwan, Ireneusz Plebankiewicz

Military Institute of Engineer Technology, Obornicka 136 Str., 50-961 Wroclaw, Poland,

**e-mail: bogdanowicz@witi.wroc.pl*

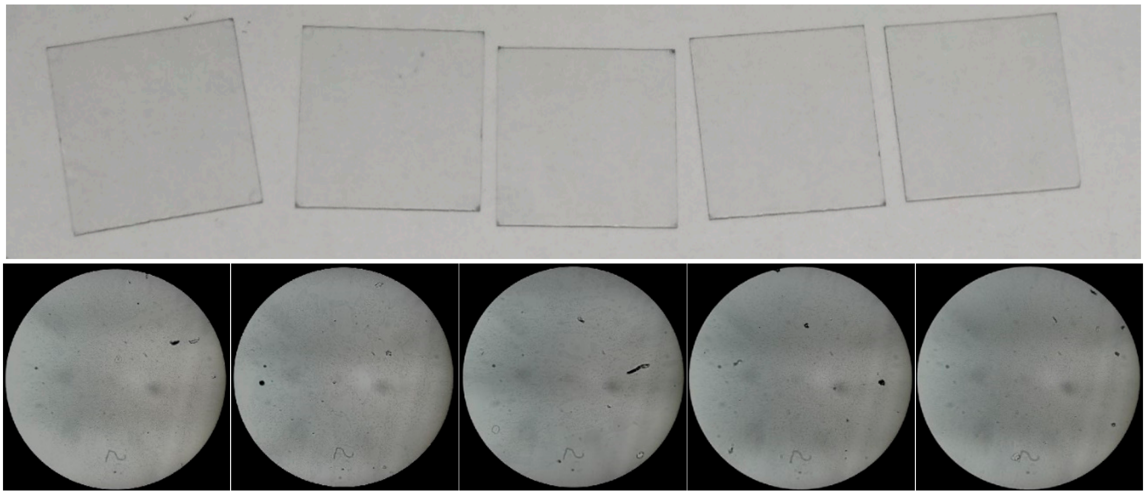

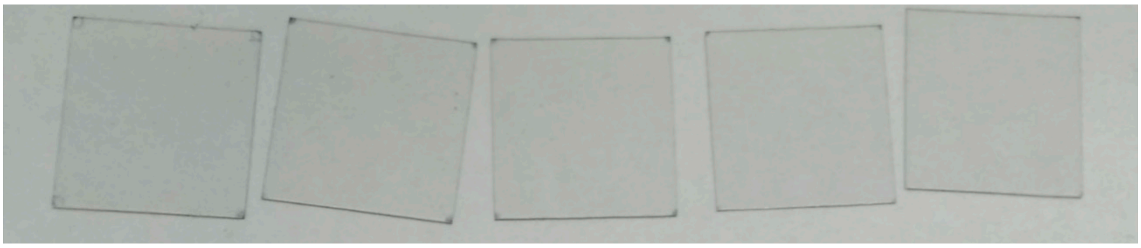

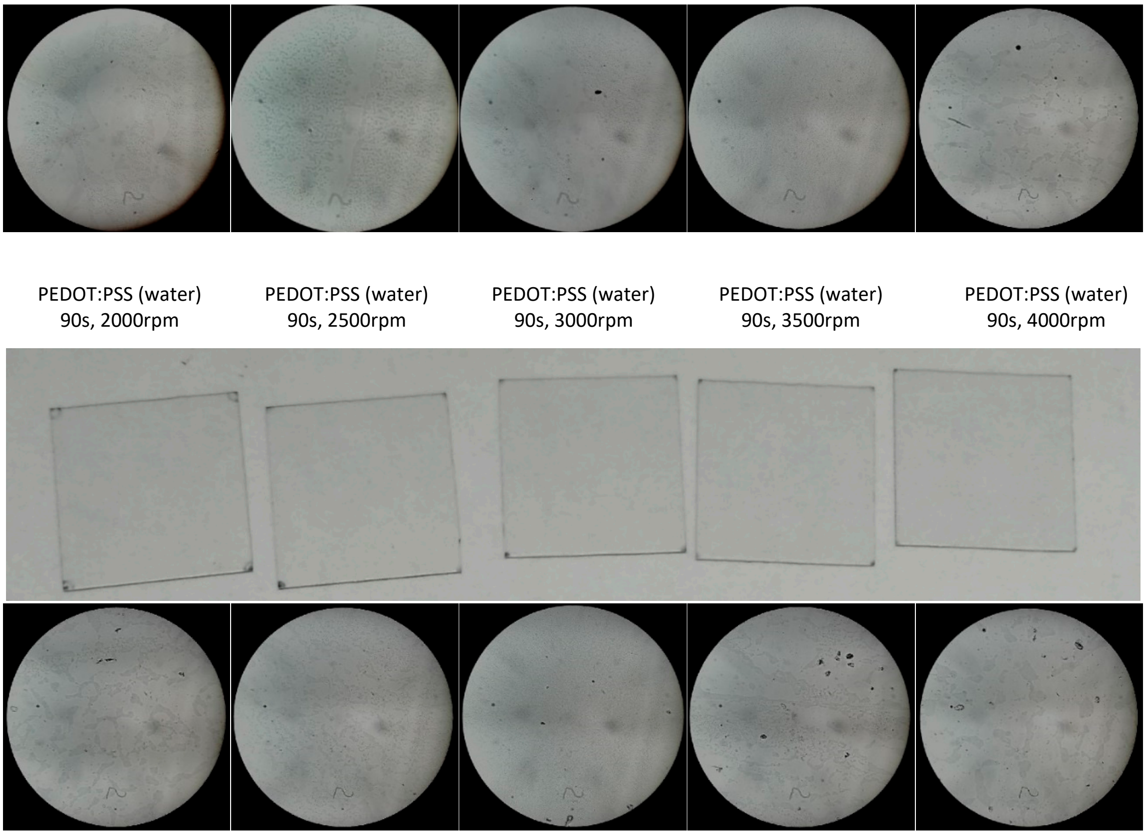

Fig. S1. Photos of PEDOT:PSS (from water) layer created with speed value from 2000 to 4000 rpm and time from 30 to 90 seconds from top to bottom along with images detected by optical microscope with magnification 18x

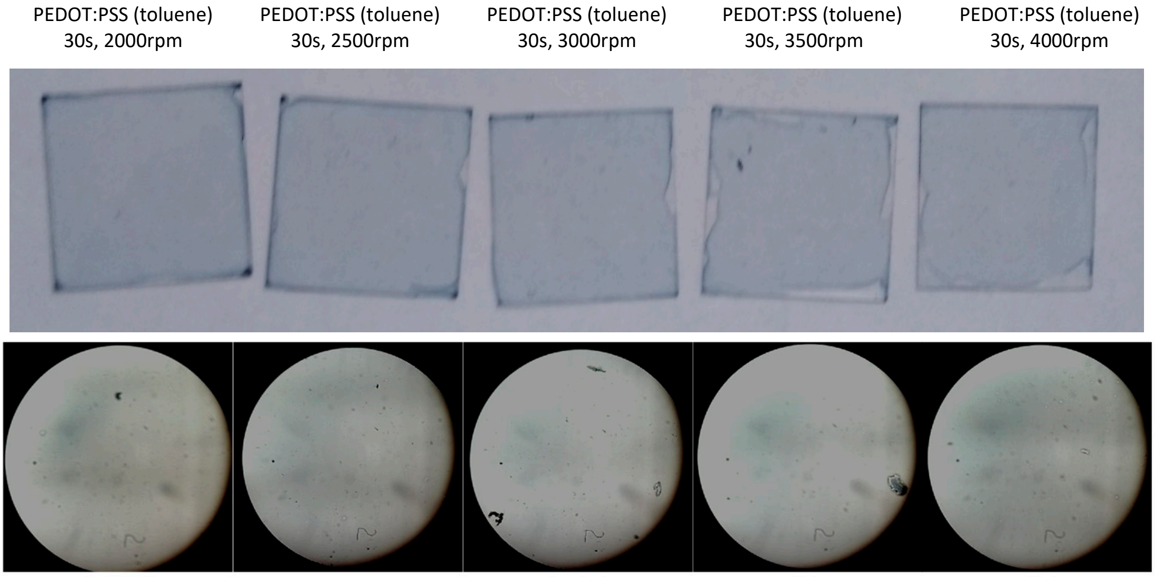

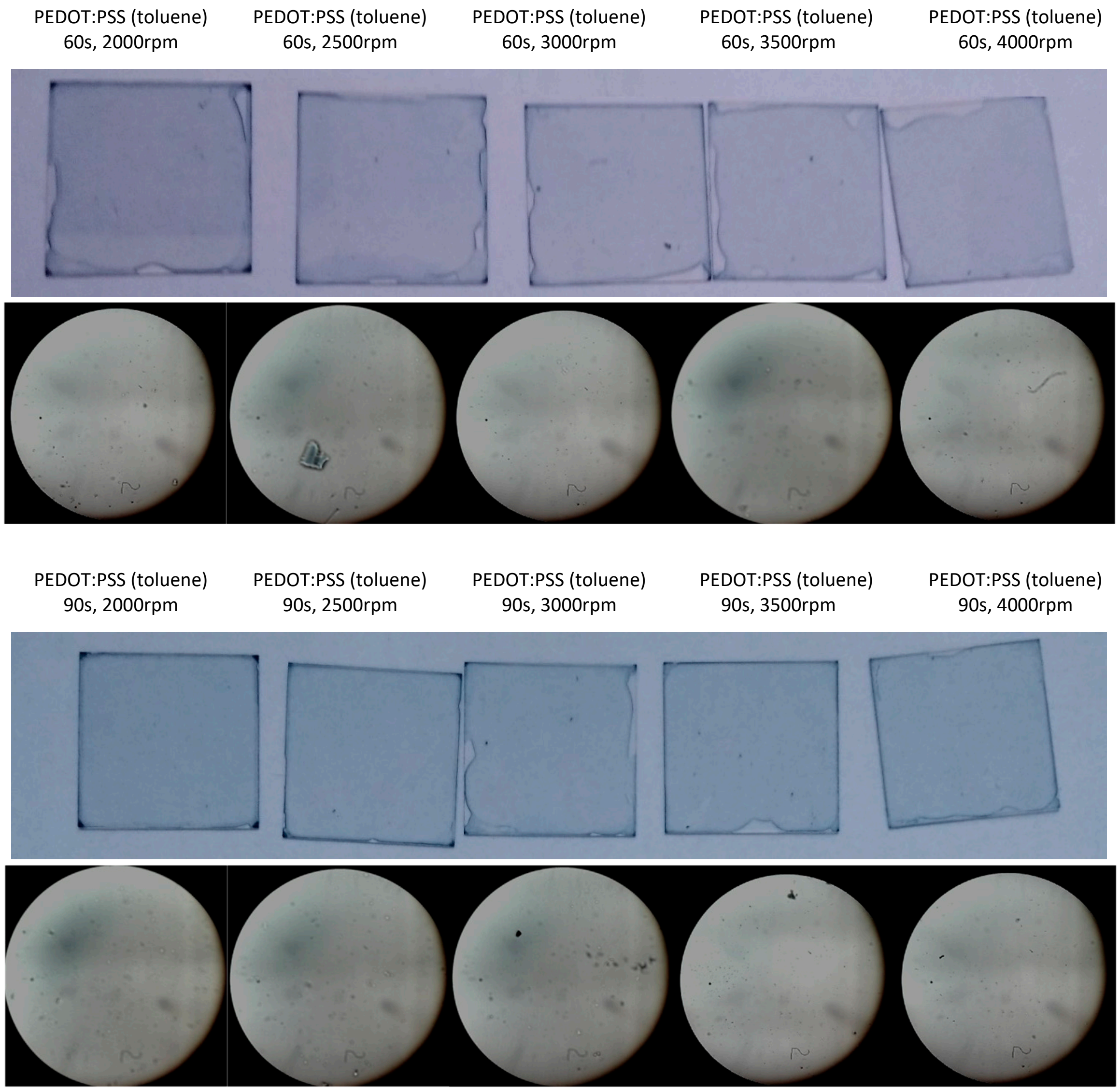

Fig. S2. Photos of PEDOT:PSS (from toluene) layer created with speed value from 2000 to 4000 rpm and time from 30 to 90 seconds from top to bottom along with images detected by optical microscope with magnification 18x

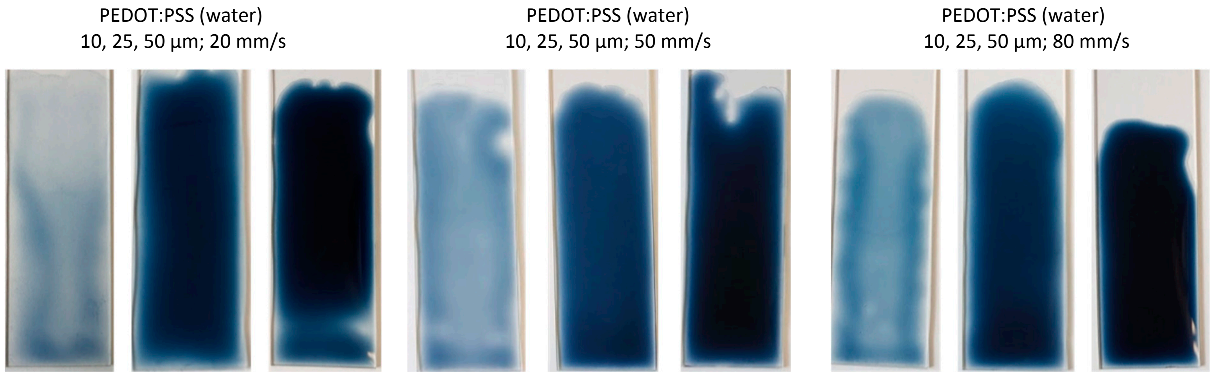

Fig. S3. Photos of PEDOT:PSS (from water) layer created with the gap set value at 10, 25, 50 µm and speed rate at 20, 50 and 80 mm/s

PEDOT:PSS (toluene)

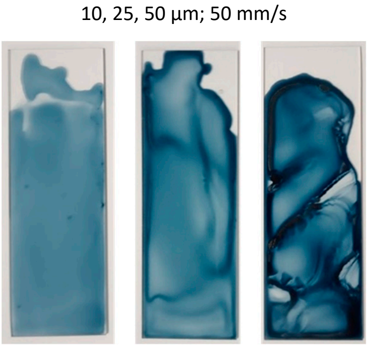

Fig. S4. Photos of PEDOT:PSS (from toluene) layer created with the gap set value at 10, 25, 50 µm and speed rate at 50 mm/s

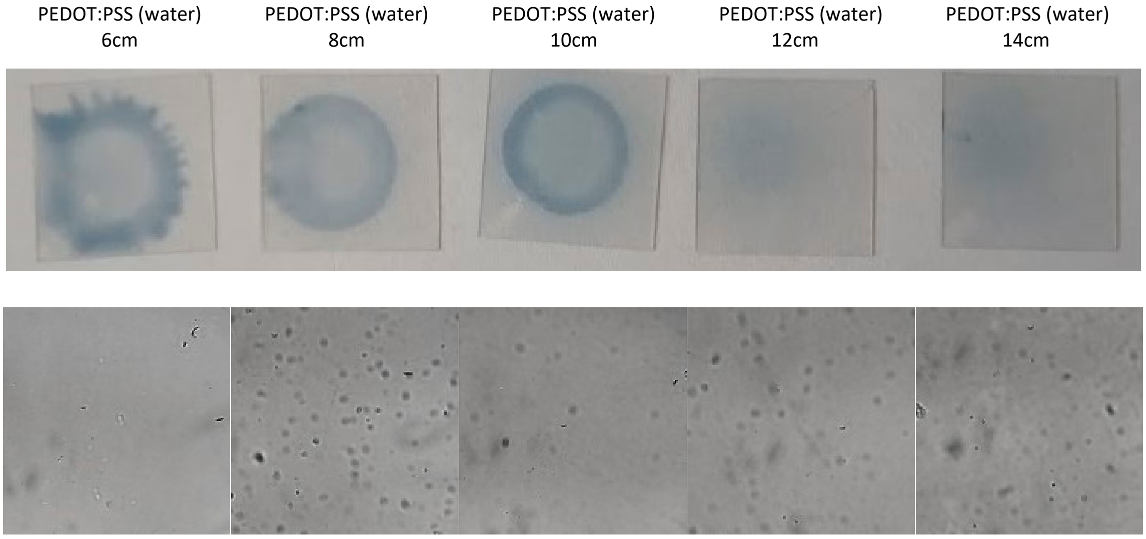

Fig. S5. Photos of PEDOT:PSS (from water) layer created by changed the distance between spray and substrate from 6 to 14 cm

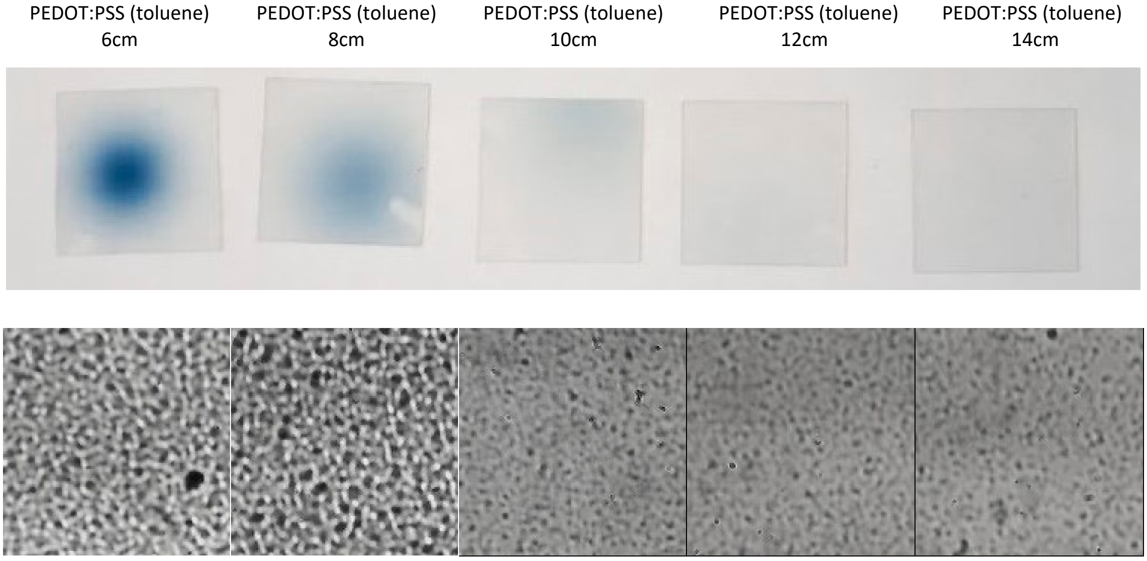

Fig. S6. Photos of PEDOT:PSS (from toluene) layer created by changed the distance between spray and substrate from 6 to 14 cm

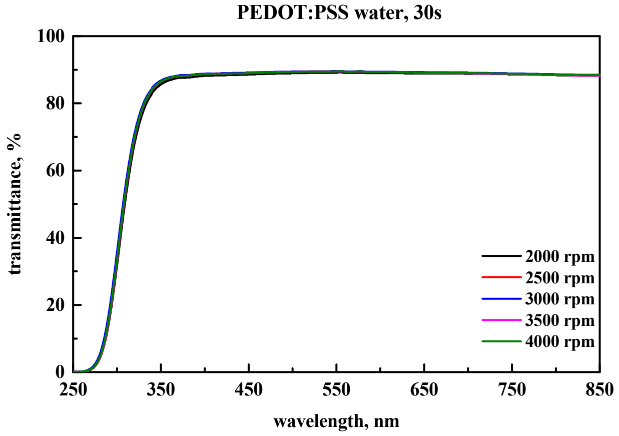

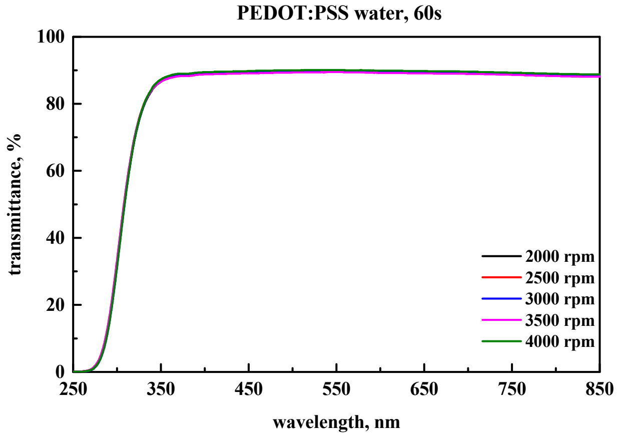

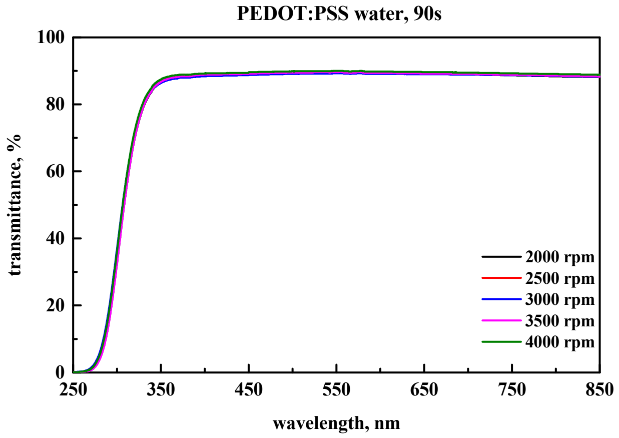

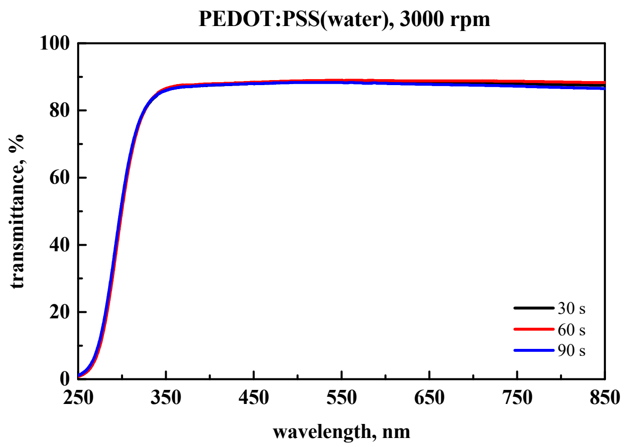

Fig. S7. UV-Vis spectra of PEDOT:PSS (from water) layers in transition mode with speed value from 2000 to 4000 rpm and time from 30 to 90 seconds from top to bottom

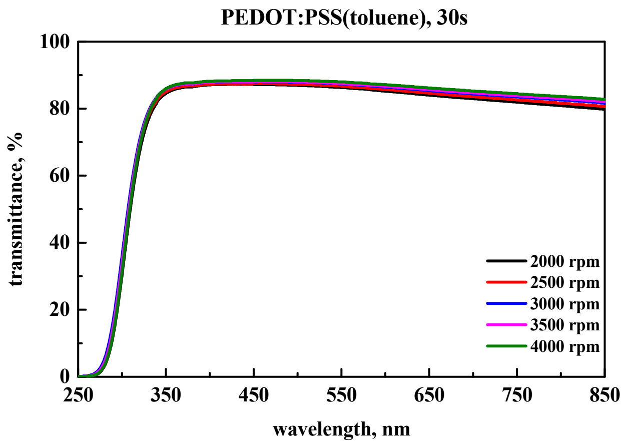

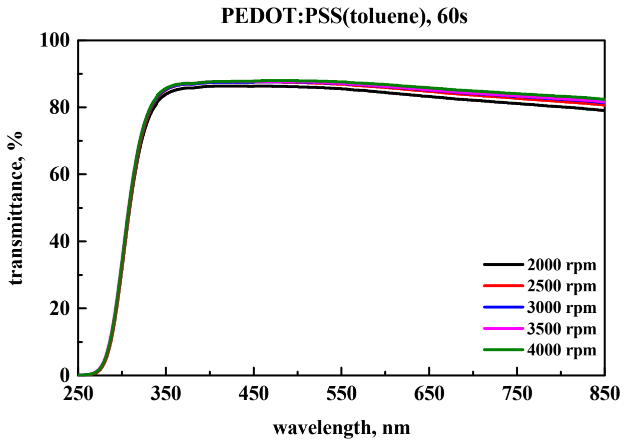

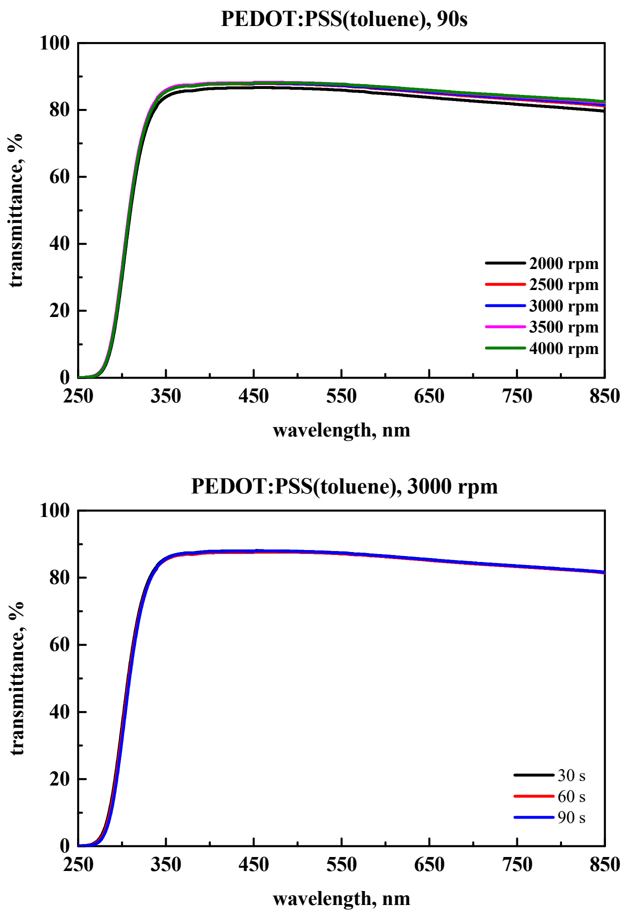

Fig. S8. UV-Vis spectra of PEDOT:PSS (from toluene) layers in transition mode with speed value from 2000 to 4000 rpm and time from 30 to 90 seconds from top to bottom
